# Mechanisms of exposure and response prevention in obsessive-compulsive disorder: effects of habituation and expectancy violation on short-term outcome in cognitive behavioral therapy

**DOI:** 10.1186/s12888-022-03701-z

**Published:** 2022-01-27

**Authors:** Björn Elsner, Tanja Jacobi, Eva Kischkel, Daniel Schulze, Benedikt Reuter

**Affiliations:** 1 Department of Psychology, Humboldt-Universität zu Berlin, Rudower Chaussee 18, 12489 Berlin, Germany; 2 Department of Psychology, Freie Universität Berlin, Habelschwerdter Allee 45, 14195 Berlin, Germany

**Keywords:** Obsessive compulsive disorder, Exposure and response prevention, Habituation, Expectancy violation, Inhibitory learning

## Abstract

**Background:**

Exposure and response prevention is effective and recommended as the first choice for treating obsessive-compulsive disorders (OCD). Its mechanisms of action are rarely studied, but two major theories make distinct assumptions: while the emotional processing theory assumes that treatment effects are associated with habituation within and between exposure sessions, the inhibitory learning approach highlights the acquisition of additional associations, implying alternative mechanisms like expectancy violation. The present study aimed to investigate whether process variables derived from both theories predict short-term outcome.

**Method:**

In a university outpatient unit, 110 patients (63 female) with OCD received manual-based cognitive-behavioral therapy with high standardization of the first two exposure sessions. Specifically, therapists repeated the first exposure session identically and assessed subjective units of distress as well as expectancy ratings in the course of exposure sessions. Based on these data, individual scores for habituation and distress-related expectancy violation were calculated and used for prediction of both percentage change on the Yale-Brown Obsessive-Compulsive Scale (Y-BOCS) and remission status after 20 therapy sessions.

**Results:**

In a multiple regression model for percentage change, within-session habituation during the first exposure was a significant predictor, while in a logistic regression predicting remission status, distress-related expectancy violation during the first exposure revealed significance. A path model further supported these findings.

**Conclusions:**

The results represent first evidence for distress-related expectancy violation and confirm preliminary findings for habituation, suggesting that both processes contribute to treatment benefits of exposure in OCD, and both mechanisms appear to be independent.

**Supplementary Information:**

The online version contains supplementary material available at 10.1186/s12888-022-03701-z.

## Background

Exposure and response prevention (ERP) is the treatment of first choice for obsessive-compulsive disorder (OCD) as evidence-based guidelines suggest [1, 2]. This is because exposure-based cognitive behavioral therapy (CBT) for OCD proved to yield symptom reduction in numerous randomized controlled trials [3, 4] and under routine care conditions [5, 6]. There is, however, a substantial number of patients who show insufficient treatment response or fail to maintain initial benefits [4, 7, 8], and it is unknown why some patients benefit and others do not. Efforts to predict treatment response by clinical or sociodemographic patient characteristics yielded inconsistent findings [9, 10]. Research on neurobiology and psychophysiology did not identify predictors for treatment response [11, 12] but rather revealed treatment-independent endophenotypes [13]. As prediction of treatment outcome by pre-treatment variables is limited, focusing on processes and mechanisms during treatment may bear good prospects for identifying additional predictors of symptom change. First approaches to predict treatment response by fear extinction learning in OCD [14] are promising but still rare. Moreover, it has been shown that frequency of and adherence to ERP [15–17] is associated with outcome, suggesting that the intervention technique itself rather than non-specific variables are crucial for symptom reduction. Nevertheless, different strategies to augment the effects of ERP, e.g. with D-cycloserine [18, 19] or motivational interviewing [20], yielded only small effects.

Further improvement appears to be impeded by insufficient knowledge on mechanisms underlying ERP [21]. Two major theories describe putative mechanisms of action, but empirical evidence is limited for both. First, emotional processing theory (EPT [22–24]) assumes that extinction of conditioned associations (such as ‘dirt – fatal disease’ turning into ‘dirt – no fatal disease’) is a key component of exposure. In addition, extinction is considered to rely on within-session and between-session habituation, i.e. the decline of fear or distress within one exposure session and across multiple sessions, respectively. Accordingly, both components are inevitable for successful ERP treatment. However, empirical research indicated that habituation may not be necessary to achieve treatment benefits [25–27]. Inhibitory learning theory (ILT [25, 28]) on the other hand, assumes that patients learn new associations during exposure (e.g., ‘dirt – no fatal disease’), which then inhibit existing associations (such as ‘dirt – fatal disease’). Although habituation may be involved, it is not considered a necessary prerequisite for learning [28]. Acquisition of new associations is rather enabled by expectancy violation, i.e. a mismatch between expectancy and outcome [28, 29]. An exposure session might thus contribute to treatment success if fear or distress constantly remains on the same level, but the experience should involve some sort of mismatch with prior expectancies or surprise. Taken together, both theories overlap in referring to Pavlovian learning as the major account of explaining effects of ERP, but they emphasize different processes as key factors of change during ERP.

Habituation and expectancy violation have been subject to a large variety of experimental laboratory studies [30–32] and clinical studies on phobias and other anxiety disorders [33–38] and evidence is mixed for both mechanisms. However, these mechanisms have rarely been investigated in clinical studies on ERP for OCD and results of previous research are conflicting. Concerning EPT, an early study by Foa, Grayson [39] investigated the relationship between treatment outcome and the decrease of Subjective Units of Distress (SUDs [40];) which served as indicator for habituation. The results suggest a relation between outcome categories (“much improved”, “improved” and “failures”) and both within-session habituation (WSH) and between-session habituation (BSH [39]) with higher levels of improvement when stronger habituation was observed. Another study found correlations between treatment outcome on the one hand and BSH indexed by both heart rate reduction across sessions and SUD reduction on the other hand [41]. Yet, WSH measured by SUDs and various physiological parameters (heart rate, skin conductance level) showed no association with treatment outcome [41]. Both early studies were limited by a small sample size (*n* = 37 and *n* = 14, respectively) and using averaged assessor ratings but no standard instruments for assessing OCD symptom severity. Taken together, early research did not consistently confirm habituation as core mechanism of action for ERP. However, recent research on contamination-based OCD with a sample of forty-one participants found within-session fear decline to be associated with post-treatment symptom reduction [42].

Other recent studies investigated habituation parameters together with mechanisms suggested by the ILT, thereby focusing on expectancy violation. In a study by Kircanski and Peris [43], treatment outcome in childhood OCD was neither consistently predicted by expectancy violation nor WSH. However, they found that greater BSH was significantly associated with improvement at mid-term assessment on the Clinical Global Impression-Improvement scale (CGI [44]) and the Children’s Yale-Brown Obsessive Compulsive Scale (CY-BOCS [45]). In line with these results, in a more recent study on childhood OCD [46], expected versus perceived post-exposure SUDs did not predict symptom reduction on the CY-BOCS. Variability in prediction accuracy (i.e., fluctuations in mismatch of actual vs. expected SUDs), however, moderated stronger OCD symptom reduction. In the two studies sample size was restricted to 35 [43] and 33 [46] participants, respectively.

In summary, surprisingly little is known about the mechanisms of action that are linked to outcome of ERP for OCD. However, theory based assumptions about mechanisms influence therapist training and impact how therapists implement ERP [26, 47, 48], entailing consequences for short- and long-term outcome [26]. For example, therapists may wonder whether it is a problem if a patient does not show habituation during ERP. Therefore, it is necessary to intensify research on mechanisms of ERP. Specifically, research on clinically defined ERP mechanisms associated with treatment outcome for OCD in adults with large sample size is missing.

The present study aimed to investigate whether and to what extent theoretically claimed mechanisms of action relate to the outcome of exposure-based CBT. Therefore, we defined selective clinical indicators of mechanisms suggested by EPT or ILT and tested their predictive value for outcome in a large sample. In line with previous studies in OCD [39, 41, 43, 46], we focused on clinical indicators of habituation as well as distress-related expectancy violation. Although expectancy violation is often measured as the discrepancy between expected and actually occurring events, we refrained from this approach for three reasons. First, many patients with OCD know that the events they fear are unlikely (e.g., fire as a consequence of not checking the stove) or do not report feared events at all (e.g., fear of touching dirty objects without expecting illness or other dangerous events, disgust, or not just right experience). Second, several concrete fears in OCD are not testable during exposure because they are long-term (e.g., “I will get cancer in ten years”) or unknowable (e.g., “I will go to hell when I die” [49]). Third, explicit testing of feared events (e.g., returning to one’s house to check whether it is on fire) can be similar to typical compulsions and thus undermine response prevention. Based on clinical characteristics of OCD (e.g., [50]) and in line with previous studies [43, 46], we assumed that distress-related expectancy violation, i.e. the discrepancy between expected and actually perceived distress, is more likely to predict outcome in OCD.

The study was implemented in the research setting of a university outpatient clinic, which combines a first treatment phase of manualized ERP-based CBT and a second phase of individually tailored CBT which is open to address other clinical problems. We hypothesized that both habituation and distress-related expectancy violation individually predict improvement at the end of the first phase, i.e. after twenty sessions. We selected this short-term outcome because its temporal proximity to the assessment of predictor variables was expected to facilitate the detection of effects. Short-term outcome was measured in terms of both symptom reduction and remission status [51], which proved to be a clinically meaningful outcome category [6].

## Methods

### Participants

**Fig. 1:**
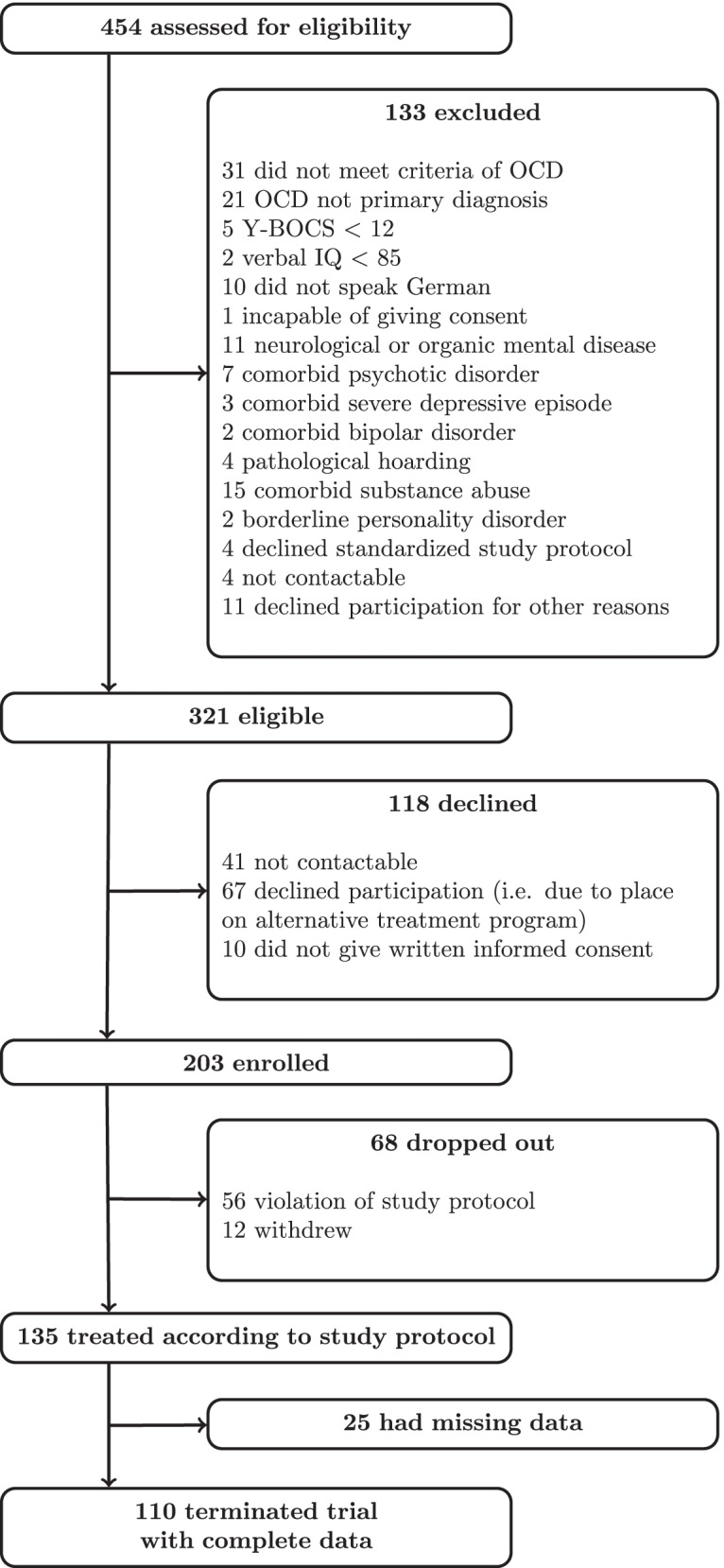
Study profile

Study participants received manual-based cognitive behavioral therapy (CBT) including exposure and response prevention at a psychological university outpatient unit based at Humboldt-Universität zu Berlin, Germany, and were admitted between April 2017 and May 2019. Referrals to the outpatient unit were made according to routine clinical care procedures. During the study period, 454 potential participants contacted the outpatient unit and 321 of them fulfilled the following inclusion criteria of the study: primary diagnosis of OCD, age between 18 and 70 years, a pre-treatment Y-BOCS-score of at least 12, and a measured verbal IQ of at least 85. Patients were excluded if they did not speak German, were not capable of giving consent, suffered from a neurological or organic mental disease, schizophrenia or another psychotic disorder, severe depressive episode, bipolar disorder, pathological hoarding, substance abuse (last three months), borderline personality disorder, or if they took benzodiazepines on a regular basis (last three months). Ten patients did not provide written informed consent and another 108 patients declined participation after admission and before the first therapy session. Of those who declined participation, 41 patients were not contactable and 67 patients declined for mostly unknown reasons; known reasons were: no more interest in participation in a research project (*n* = 2); no more motivation for engaging in CBT (*n* = 3); patients did not see indication for therapy any longer or do not suffer from symptoms anymore (*n* = 2); inpatient treatment (*n* = 6); found another therapy placement (*n* = 6); moved to another city (*n* = 2). Thus, 203 patients participated in the study, but for 56 of them the study protocol was violated (*n* = 12 did not meet the time criterion to terminate the first phase of treatment comprising 20 manualized sessions within maximally 14 weeks, *n* = 20 did not meet criteria for time interval between the first two ERP exercises, for *n* = 22 therapists did not provide complete formal adherence checklists, *n* = 1 interrupted treatment due to inpatient admission, *n* = 1 did not want to engage in exposure therapy; see Treatment and Study Protocol) and another twelve patients did not complete therapy. 135 patients received treatment according to the study protocol. 25 of them had missing data either in the primary outcome variable or in one of the exposure process variables. Therefore, 110 patients terminated the trial with complete data (Fig. 1). The final sample (*n* = 110) and the sample of participants enrolled but not included in the final sample (*n* = 93) did not differ regarding demographic or clinical characteristics, however, participants in the final sample had significantly more often comorbid mental disorders (Table 1).

**Table 1:** Group differences in demographic and clinical variables of the final sample (*n* = 110) and enrolled participants who were excluded (*n* = 93) at admission (t_0_)

	final sample		enrolled but excluded		
Variable	*n*		*n*		Test
Gender	110	63 f / 47 m	92	47 f / 45 m	*p* = .398
Comorbidity of mental disorder	110	88	93	61	*p* = .026
Psychotropic medication	110	62 none / 48 at least one	93	54 none / 39 at least one	*p* = .887
		M (SD)		M (SD)	
Age (years)	110	33.8 (10.8)	92	32.7 (10.0)	*t*(197.83) = 0.74, *p* = .463
Socioeconomic status	110	9.7 (3.8)	87	9.9 (3.7)	*t*(186.49) = −0.30, *p* = .761
Y-BOCS	110	23.4 (4.4)	91	23.3 (5.2)	*t*(177.85) = 0.17, *p* = .862
OCI-R	110	29.9 (13.2)	91	28.7 (11.8)	*t*(197.9) = 0.70, *p* = .484
MADRS	110	13.1 (7.0)	91	13.7 (9.0)	*t*(167.93) = −0.49, *p* = .624
BDI-II	110	19.5 (9.9)	91	18.6 (10.2)	*t*(189.54) = 0.60, *p* = .547
BSI-GSI	110	1.04 (0.54)	91	0.97 (0.59)	*t*(185.39) = 0.93, *p* = .356
GAF	110	56.0 (8.4)	89	54.5 (8.4)	*t*(188.73) = 1.25, *p* = .213

*Note*. Comorbidity of mental disorder = at least one; Y-BOCS = Yale-Brown Obsessive-Compulsive Scale interview score; OCI-R = Obsessive Compulsive Inventory - Revised; MADRS = Montgomery-Åsberg Depression Rating Scale; BDI-II = Beck Depression Inventory II; BSI-GSI = Global Severity Index of the Brief Symptom Inventory; GAF = Global Assessment of Functioning; Socioeconomic status was assessed according to [52]

The final sample (*n* = 110, 63 female) had a mean age of 33.8 years (SD = 10.8). Eighty-eight (80.0%) of them suffered from at least one comorbid mental disorder. Most common diagnoses were current or remitted affective disorders and anxiety disorders (Supplementary Table 1). At the time of admission, 62 participants (56.4%) were free of psychotropic medications and 48 participants (43.6%) took at least one psychotropic medication. Most common medications were selective serotonin reuptake inhibitors (SSRIs) and other antidepressants (Supplementary Table 1). During the study period, most medicated participants were medication stable (*n* = 40) and few discontinued medication (*n* = 8). Seven participants, who were unmedicated at admission, started medication during the study period.

The study protocol was approved by the local review board of Humboldt-Universität zu Berlin (protocol number 2016-33) and met the criteria of the revised Declaration of Helsinki. All study participants provided written informed consent.

### Clinical assessment

Routine assessment at admission (t_0_) included the German version of the Structured Clinical Interview for DSM-IV mental disorders and personality disorders [53, 54], the Yale-Brown Obsessive-Compulsive Scale interview (Y-BOCS [55]), the Montgomery-Åsberg Depression Rating Scale (MADRS [56]), and the Global Assessment of Functioning (GAF [57]). Additionally, the Obsessive Compulsive Inventory - Revised (OCI-R [58]), the Beck Depression Inventory II (BDI-II [59]), the Brief Symptom Inventory (BSI [60]), and a Y-BOCS-self-rating version [61] were administered as self-rating questionnaires. Y-BOCS interview, Y-BOCS self-rating scale, OCI-R, MADRS, BDI-II, BSI, and GAF were repeated at the time of the first therapy session (t_1_) and after the twentieth therapy session (t_20_) in order to assess the course of obsessive-compulsive, depressive and general psychological symptoms, respectively. To check whether OCD symptom severity already changed prior to the first exposure, the Y-BOCS self-rating scale was additionally assessed immediately before the first EPR session (t_ERP1_).

All interviews at all assessment points were conducted by trained clinical psychologists who were not involved in treatment.

### Treatment and study protocol

Treatment was delivered by 21 clinical psychologists (diploma or masters degree) who had additional two- to five years formal training in CBT and most of them were licensed psychotherapists according to German psychotherapy law. Treatment consisted of a first phase with a largely standardized, manual-based procedure optimized to meet study requirements (internally devised lab manual based on [62, 63]), and a second phase of individually tailored CBT, which allowed addressing individual needs like continuing ERP treatment or addressing comorbid disorders. Treatment termination was based on clinical decisions, so that total treatment duration was variable. For the present analyses, we chose to predict the end of the first phase (after 20 sessions), because this period comprised homogenous ERP procedures. Moreover, we expected to increase the chance of detecting process-outcome effects if the temporal relationship between predictors and outcome variables is close and uniform for all patients. Post-treatment outcomes (after termination of phase 2) are still collected and are not analyzed for the purpose of this paper.

The first phase comprised 20 therapy sessions (50 min each) with face-to-face consultations twice a week. In session one through eight, mandatory manual contents were psychoeducation, defining individual therapy goals, and conveying an ERP rationale on the basis of a cognitive-behavioral OCD model emphasizing the role of negative reinforcement and prevention of corrective experience by avoidance and compulsions. Therapists were instructed to refer neither to habituation nor to expectancy violation as possible mechanisms of action. Session nine to 20 were conducted as double sessions with a total duration of 100 min, including at least four therapist-guided ERP exercises that followed a gradual, hierarchically-driven course. In addition, therapist and patient planned, analyzed and monitored self-guided ERP exercises (conducted between sessions) and response prevention in daily routine. Phase 1 had to be terminated after a maximum duration of 14 weeks. Therapists indicated adherence to the study protocol by formal checklists where accomplished elements of therapy were recorded after each session.

Specifically, the first two ERP exercises were highly standardized: The first exposure task was repeated identically in the second session within one to four days. Therefore, assessed exposure process parameters are likely to be comparable between the two sessions. The first ERP exercise was conducted on average at the eighth session, but the actual session number varied across participants (range 5–15). The exposure task had a medium level of difficulty as indicated by the participant prior to the first exposure session. In order to create this individual difficulty level, participants ordered different symptom-eliciting situations hierarchically ranging from 0 (“not difficult at all”) to 10 (“highest imaginable difficulty”). Medium difficulty was defined across participants by a level of 4–6. The level of difficulty was not changed during the two standardized exercises, which always lasted exactly 45 min. Participants were excluded from the study (*n* = 15) in case no or short fear levels during the exercise impeded conducting ERP for the entire duration as this was a failure to comply with the study protocol. In these cases, the exercise was terminated prematurely if an a priori defined cutoff criterion (SUDs of 0 or 1 over a period of at least 15 min) was met. Both ERPs were terminated according to the study protocol regardless of the fear levels at the end of the session. No homework or self-guided exposure was assigned between the first two ERPs. Therapists recorded several data during both EPRs on a protocol sheet: immediately before conducting the exposure task, participants rated on a 0 to 10 Likert scale what they expected to be (1) the highest subjective level of fear or distress [0 = none, 10 = highest imaginable]; [40] during the ERP task, and (2) the level of fear or distress at the end of the session (after 45 min). Moreover, therapists assessed (3) the pre-exposure level of confidence in conducting the exposure task as planned (0 = not confident at all, 10 = most confident). During ERP, therapists asked participants (4) to rate their SUDs every three minutes (minute 0 through 45) on a 0 to 10 Likert scale. Immediately after the end of the ERP task, therapists recorded (5) the participant’s rating on how high fear or distress was during ERP compared to their expectancy prior to ERP on a 0 to 10 Likert scale (0 = much less than expected, 5 = as expected, 10 = much higher than expected), thus assessing a direct self-rating for expectancy violation in both ERP sessions (EVself_ERP1_, EVself_ERP2_). Moreover, (6) participant’s post-session confidence in conducting the same exposure task again was recorded on a 0 to 10 Likert scale.

### Exposure process variables

#### Within-session habituation

We calculated a difference score between the maximum SUD level and the ensuing minimum SUD level during the ERP exercise (4) in order to assess within-session habituation (WSH). A comparable operationalization was applied before by Foa, Grayson [39] using the change between the highest and the following lowest anxiety level of the same session, while other studies calculated WSH as the difference between the maximum score and the final score at the end of exposure (e.g., [64]). In the present study, the minimum SUD rating corresponded to the final SUD rating for 79 patients (71.8%) and was lower than the final rating for 31 patients (28.2%) in exposure 1. During exposure 2, minimum and final score were equal for 82 patients (74.5%) and minimum scores were lower than the final rating for 28 patients (25.5%). As proposed by Kircanski and Peris [43], we applied a continuous measure of SUD levels in order to examine “more nuanced fluctuations in distress” [43], and therefore it was possible to determine the individual minimum following the maximum SUD level. Thus, greater difference scores represented stronger habituation (minimum score subtracted from maximum score). The difference score was applied to the first two standardized ERP sessions, resulting in individual parameters for both sessions (WSH_ERP1_, WSH_ERP2_). While the difference score may be an easy and intuitive way of approximating WSH, the course of SUDs over time might not be represented appropriately by this score. As WSH was continuously measured during the two standardized ERP sessions, more than two data points were available and it was possible to model individual slopes across the SUD scores of an exposure as an alternative predictor of outcome. This was not possible for all other exposure process variables because only two data points (e.g. prior distress-related expectancy vs. final SUDS score) were available. We calculated individual linear slope parameters for each participant using R linear mixed-effects models package nlme [65] in order to create growth curves with random intercepts and random slopes for SUDs over time. Negatives slopes represented higher SUD levels at the beginning than at the end of exposure. Again, linear slopes were calculated for the first two standardized exposure sessions (Slope_ERP1_, Slope_ERP2_).

#### Between-session habituation

As an indicator for between-session habituation (BSH) we calculated the SUD reduction from the first to the second standardized ERP regarding their maximum scores during the exposure task (4). Thus, higher scores represent stronger SUD reductions.

#### Expectancy violation towards the maximum SUD score

In order to assess distress-related expectancy violation regarding the highest SUD level during ERP, we calculated a difference score between the prior expectation towards the maximum SUD level (1) and the real maximum SUD level during the exposure task (4) for the first two standardized EPR sessions (EVmax_ERP1_, EVmax_ERP2_). Positive scores indicated higher expected maximum SUD levels than experienced maximum SUD levels (overestimation of fear) and negative scores indicated lower expected than real maximum SUD levels (underestimation of fear).

#### Expectancy violation towards the end SUD score

Distress-related expectancy violation regarding the SUD level at the end of ERP (after 45 min) was assessed by a difference score between the prior expectation towards the end SUD level (2) and the experienced SUD level at the end of the exposure task (4) for the first two standardized EPR sessions (EVend _ERP1_, EVend _ERP2_). Positive scores indicated higher expected end SUD levels than experienced end SUD levels (overestimation of fear) and negative scores indicated lower expected than experienced end SUD levels (underestimation of fear).

#### Direct self-rating of expectancy violation towards the maximum SUD score

This measure was assessed immediately after the two standardized ERPs (5), directly resulting in two exposure process variables (EVself_ERP1_, EVself_ERP2_). In these variables, higher scores indicated higher than expected SUDs. As this was inverse to the direction in EVmax and EVend, where higher scores indicated lower than expected SUD scores, all EVself scores were inverted (i.e., multiplied by − 1).

#### ERP-related self-efficacy change

As it is possible that expectancy changes during ERP may also relate to beliefs about prospective events, former research recommended to assess coping self-efficacy [46]. Moreover, van Hout and Emmelkamp [36] found a relation between overestimation of the level of distress during exposure and subsequently increased self-efficacy. There is also first evidence that self-efficacy mediates outcome of self-guided ERP [17, 66]. In order to account for self-efficacy change (SEC) as a control variable in the present study, we calculated a difference score between the confidence in conducting exposure as planned prior to ERP (3) and the post-ERP confidence in conducting the same ERP task again (6) for the first two standardized ERP sessions (SEC_ERP1_, SEC_ERP2_). Higher sores indicated an increase in self efficacy from pre- to post-exposure assessment.

### Primary outcome variables

Using the exposure process variables, we predicted short-term outcome after twenty therapy sessions (t_20_) by the time of termination of the manual-based treatment, i.e. the first phase. Primary outcome variables were (a) the percentage change of the Y-BOCS interview scores and (b) the achievement of remission status from the first (t_1_) to the last (t_20_) manual-based treatment session. Remission was defined according to international expert consensus criteria [Y-BOCS total score ≤ 12; [51] without applying the CGI Improvement scale [6, 67].

### Data analysis

Data was analyzed using R version 3.5.1. While multiple regression is appropriate in order to predict metric outcomes, logistic regression can be used to predict categorial data. Thus, a multiple regression model served to predict the percentage change of the Y-BOCS interview score and a logistic regression model was calculated to predict remission status, respectively. In both regression models, the Y-BOCS score assessed at t_1_ was included in order to control for pretreatment symptom severity. Due to the pilot character of the study, no power analyses were conducted before the study, but post-hoc analyses showed that the final sample size allowed to detect the hypothesized effects with a power of 96.7% (multiple regression) and 99.7% (logistic regression), respectively. We further repeated both regression models including a variable indicating medication during the study period (*n* = 47) versus no medication. In a second step, we selected significant predictors from the regressions and applied R package lavaan [68] to calculate a path model accounting for the temporal sequence of the assessed variables. Two further exploratory analyses were conducted. First, we explored whether the values of significant predictors differed between remitters and non-remitters. Second, we investigated whether the theoretically distinct self-report measures actually reflected the same psychological construct. Hence, we conducted a confirmatory factor analysis (CFA) in order to evaluate whether parameters of habituation and expectancy violation reflect a single underlying dimension.

## Results

### Treatment outcome after 20 sessions

None of the participants terminated treatment before the end of the manual-based treatment phase, i.e. until t_20_. However, twelve patients terminated treatment after only five or less additional sessions. Seventeen patients (15.5%) reached remission status (Y-BOCS ≤ 12) until t_20_.

**Fig. 2:**
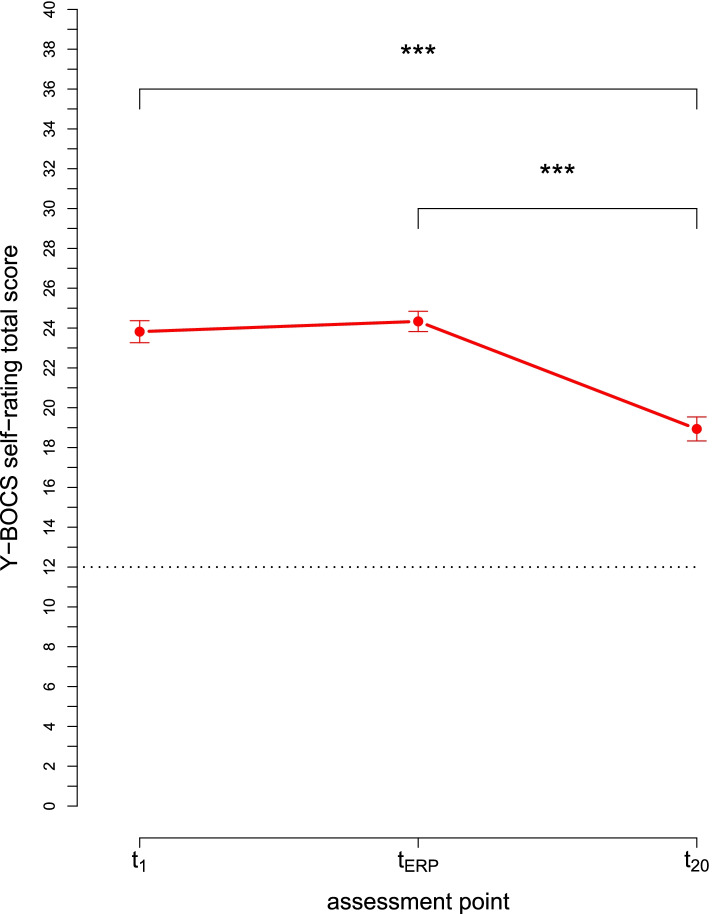
Mean symptom change on the Y-BOCS self-rating from the time of the first therapy session (t_1_) across the time immediately before the first ERP (t_ERP_) to the time after 20 sessions (t_20_). Error bars indicate standard errors. *Note*. n(t_1_) = 105, n(t_ERP_) = 106, n(t_20_) = 105

Average Y-BOCS interview scores significantly reduced from t_1_ to t_20_ with a large effect size (Table 2). Considering secondary outcome variables for OCD symptoms, average Y-BOCS-self-rating scores reduced across the assessment points, *F*(1.79,176.95) = 77.45, *p* < .001 (Fig. 2). On group level, self-rated OCD symptoms were not reduced significantly from t_1_ to the time immediately before the first ERP (t_ERP_), *t*(206.68) = − 0.68, *p* = .496. However, there was a significant mean symptom reduction from t_ERP_ to t_20_, *t*(203.82) = 6.84, *p* < .001 and from t_1_ to t_20_, *t*(207.70) = 5.97, *p* < .001 (Fig. 2). Moreover, we observed a significant mean reduction on the OCI-R from t_1_ to t_20_ (Table 2).

**Table 2:** Course of mean symptom scores from the time of the first therapy session (t_1_) to the time after 20 sessions (t_20_)

					t_1_-t_20_		
Measure	n t_1_	M t_1_ (SD)	n t_20_	M t_20_ (SD)	t	(df)	d
Y-BOCS	110	24.2 (5.2)	110	18.5 (5.9)	10.49***	(109)	1.02
OCI-R	110	27.6 (12.7)	110	21.1 (12.5)	9.96***	(109)	0.52
MADRS	110	10.2 (7.3)	110	9.2 (7.6)	1.59	(109)	0.13
BDI-II	110	16.5 (10.6)	110	12.8 (9.8)	6.13***	(109)	0.36
BSI-GSI	110	0.92 (0.59)	110	0.76 (0.57)	5.14***	(109)	0.27
GAF	103	54.3 (10.8)	104	61.1 (12.4)	−4.22***	(201.69)	0.59

*Note*. Y-BOCS = Yale-Brown Obsessive-Compulsive Scale interview score; OCI-R = Obsessive Compulsive Inventory - Revised; MADRS = Montgomery-Åsberg Depression Rating Scale; BDI-II = Beck Depression Inventory II; BSI-GSI = Global Severity Index of the Brief Symptom Inventory; GAF = Global Assessment of Functioning; d = Cohen’s d with pooled standard deviations; *** *p* < .001

Also, depressive symptoms reduced significantly from t_1_ to t_20_ as indicated by the BDI-II. However, MADRS mean scores did not reflect improvement from t_1_ to t_20_, (Table 2). General psychological symptoms as indicated by the Global Severity Index of the BSI were also significantly reduced from t_1_ to t_20_ (Table 2), and the GAF increased significantly from t_1_ to t_20_ (Table 2).

### Correlations

Intercorrelations among exposure process variables were predominantly small to moderate, see Supplementary Table 2. Correlations between exposure process variables and primary outcome variables are shown in Table 3. Notably, WSH, EVmax and EVend from ERP1 correlated significantly positive with the percentage change of the Y-BOCS score from t_1_ through t_20_, whereas no significant association could be observed for variables from ERP2. Regarding remission status, the only significant correlation emerged with EVend of ERP1.

**Table 3:** Correlations of exposure process variables with outcome variables

	Y-BOCS score change (%)	Remission status
WSH_ERP1_	.26**	.12
WSH_ERP2_	.01	−.03
BSH	.05	.08
EVmax_ERP1_	.20*	.11
EVmax_ERP2_	.06	−.03
EVend_ERP1_	.27**	.28**
EVend_ERP2_	−.03	−.14
EVself_ERP1_	.14	.02
EVself_ERP2_	−.08	−.18
SEC_ERP1_	.06	−.07
SEC_ERP2_	.09	.01

*Note*. Y-BOCS = Yale-Brown Obsessive-Compulsive Scale interview; ERP1 = first standardized exposure with response prevention; ERP2 = second standardized exposure with response prevention; WSH = within-session habituation; BSH = between-session habituation; EVmax = expectancy violation towards the maximum SUD score; EVend = expectancy violation towards the end SUD score; EVself = direct self-rating of expectancy violation towards the maximum SUD score; SEC = self efficacy change; * *p* < .05; ** *p* < .01

### Prediction of percentage change of the Y-BOCS score

The multiple linear regression model with habituation and distress-related expectancy violation variables predicting the percentage change of the Y-BOCS interview score was significant overall, *F*(12,97) = 2.24, *p* = .015, *R*^*2*^ = .217, adjusted *R*^*2*^ = .120. Apart from the Y-BOCS score at t_1_, the only significant predictor was within-session habituation during the first ERP (Table 4). Repeating the regression with medication status during the study period did not change the pattern of results and medication was not a significant predictor, *β* = − 0.04, *p* = .651. An alternative model with WSH being estimated by random intercepts and random linear slopes for time across the course of SUDs during ERP was also significant, *F*(12,97) = 2.32, *p* = .012, *R*^*2*^ = .223, adjusted *R*^*2*^ = .127, and yielded the same pattern of results (Supplementary Table 3). The two different WSH estimations correlated negatively (ERP1 *r* = −.61, ERP2 *r* = −.71), because negative slopes indicate a higher reduction of SUDs across time.

**Table 4:** Multiple regression model predicting percentage change of the Y-BOCS score from t_1_ to t_20_

Coefficient	B (SE)	β	95% CI for β	p	partial η^2^
Constant	−9.26 (15.34)				
Y-BOCS score t_1_	0.86* (0.43)	0.19	0.01–1.70	.046	.040
WSH_ERP1_	3.98* (1.84)	0.31	0.34–7.62	.033	.046
EVmax_ERP1_	3.40 (1.99)	0.22	−0.55 - 7.34	.091	.029
EVend_ERP1_	0.81 (1.13)	0.09	−1.46 - 3.08	.479	.005
EVself_ERP1_	−0.22 (1.35)	−0.02	−2.90 - 2.46	.872	.000
SEC_ERP1_	0.12 (1.15)	0.01	−2.17 - 2.41	.917	.000
WSH_ERP2_	−2.00 (1.88)	−0.16	−5.73 - 1.72	.289	.012
EVmax_ERP2_	1.31 (2.03)	0.09	−2.72 - 5.33	.521	.004
EVend_ERP2_	−0.58 (1.43)	−0.06	−3.42 - 2.25	.684	.002
EVself_ERP2_	−1.18 (1.43)	−0.09	−4.03 - 1.66	.411	.007
SEC_ERP2_	2.52 (1.57)	0.15	−0.61 - 5.64	.113	.026
BSH	−0.06 (2.19)	−0.00	−4.40 - 4.27	.977	.000

*Note*. Y-BOCS = Yale-Brown Obsessive-Compulsive Scale interview; ERP1 = first standardized exposure with response prevention; ERP2 = second standardized exposure with response prevention; WSH = within-session habituation; BSH = between-session habituation; EVmax = expectancy violation towards the maximum SUD score; EVend = expectancy violation towards the end SUD score; EVself = direct self-rating of expectancy violation towards the maximum SUD score; SEC = self efficacy change; *p < .05

### Prediction of remission status

The logistic regression model predicting remission status after 20 therapy sessions with the same set of exposure process variables was significant, *Χ*^*2*^(12) = 39.50, *p* < .001, Nagelkerke *R*^*2*^ = .523. As opposed to the multiple regression model, parameters for WSH did not predict remission status. Distress-related expectancy violation towards the end SUD score (EVend), however, predicted remission status significantly (Table 5). Interestingly, EVend_ERR1_ predicted remission positively (Odds Ratio, OR = 2.03) while EVend_ERP2_ revealed a negative relationship with remission at t_20_ (OR = 0.60), indicating that EVend had opposite effects in the two different exposure sessions. Repeating the regression with medication status during the study period did not change the pattern of results and medication was not a significant predictor, *OR* = 1.74 (*CI* 0.38–8.22), *p* = .471. Using random effects linear slopes instead of original WSH parameters also yielded a significant logistic regression model, *Χ*^*2*^(12) = 40.92, *p* < .001, Nagelkerke *R*^*2*^ = .538, but did not change the pattern of results (Supplementary Table 4).

**Table 5:** Logistic regression model predicting remission status at t_20_

Coefficient	B (SE)	exp b (OR)	95% CI for OR	p
Constant	1.15 (2.75)			.676
Y-BOCS score t_1_	−0.26*** (0.08)	0.77	0.65–0.88	< .001
WSH_ERP1_	−0.27 (0.33)	0.76	0.38–1.42	.407
EVmax_ERP1_	0.03 (0.42)	1.03	0.43–2.38	.950
EVend_ERP1_	0.71** (0.27)	2.03	1.30–3.75	.008
EVself_ERP1_	−0.08 (0.26)	0.92	0.55–1.56	.760
SEC_ERP1_	−0.18 (0.27)	0.84	0.47–1.34	.497
WSH_ERP2_	0.20 (0.28)	1.23	0.70–2.11	.481
EVmax_ERP2_	0.53 (0.39)	1.70	0.85–4.00	.174
EVend_ERP2_	−0.52* (0.26)	0.60	0.34–0.95	.045
EVself_ERP2_	−0.40 (0.26)	0.67	0.38–1.10	.129
SEC_ERP2_	0.32 (0.26)	1.38	0.84–2.43	.207
BSH	0.40 (0.39)	1.50	0.69–3.26	.295

*Note*. OR = Odds Ratio; Y-BOCS = Yale-Brown Obsessive-Compulsive Scale interview; ERP1 = first standardized exposure with response prevention; ERP2 = second standardized exposure with response prevention; WSH = within-session habituation; BSH = between-session habituation; EVmax = expectancy violation towards the maximum SUD score; EVend = expectancy violation towards the end SUD score; EVself = direct self-rating of expectancy violation towards the maximum SUD score; SEC = self efficacy change; * *p* < .05; ** *p* < .01; *** *p* < .001

### Path model

**Fig. 3:**
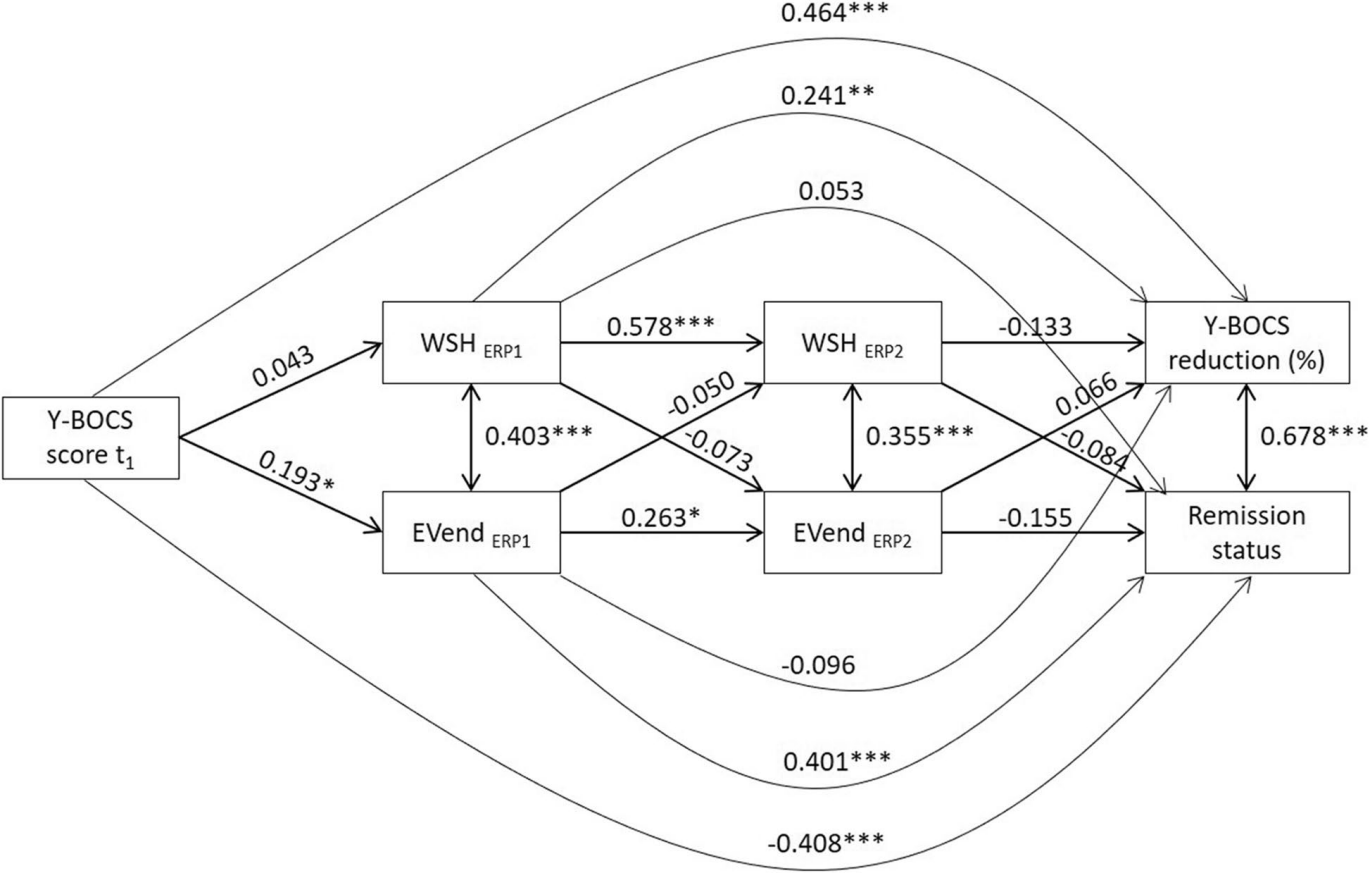
Path model predicting percentage change of the Y-BOCS score from t_1_ to t_20_ and remission status at t_20_. *Note*. Y-BOCS = Yale-Brown Obsessive-Compulsive Scale interview; ERP1 = first standardized exposure with response prevention; ERP2 = second standardized exposure with response prevention; WSH = within-session habituation; EVend = expectancy violation towards the end SUD score; * *p* < .05; ** *p* < .01; *** *p* < .001

As the regression models revealed within-session habituation and distress-related expectancy violation towards the end SUD score to be significant predictors for outcomes, we included both exposure process variables in a path model predicting percentage change of the Y-BOCS score and remission status at t_20_ (Fig. 3). This model included the same variables as the regression model but enabled us to put them into an appropriate temporal order. All variables predicted the final outcomes (remission and reduction), but all previous time points were only predicted by directly preceding measurements to model a hypothetical causal flow. The model fit was adequate as reflected by several fit indices; *χ*^*2*^(2) = 1.17, *p* = .556; *CFI* = 1.00; *RMSEA* = 0.00; *SRMR* = 0.02 [69]. In accordance with the regression models, a significant direct pathway from WSH_ERR1_ to percentage change on the Y-BOCS emerged; and EVend_ERR1_ predicted remission status. In conclusion, the effects found in the regression analysis still held when controlling for their temporal succession.

### Exploratory analyses

While remitters and non-remitters did not differ in within-session habituation during the first ERP (*t*(21.13) = 1.13, *p* = .272), remitters showed significantly stronger distress-related expectancy violation towards the end SUD level of the first ERP (*M* = 1.76, overestimation of fear) compared to non-remitters (*M* = − 0.22, underestimation of fear), *t*(21.2) = 2.89, *p* = .009.

Conducting confirmatory factor analysis on the variables WSH_ERP1_, BSH, EVmax_ERP1_, EVend_ERP1_, EVself_ERP1_ and SEC_ERP1_ revealed that a one-dimensional CFA model yielded an inadequate fit (*χ*^*2*^(9) = 39.34, p < .001, *CFI* = 0.66, *RMSEA* = 0.18, *SRMR* = 0.11) with significant loadings only for parameters of expectancy violation (Supplementary Table 5).

## Discussion

This study aimed to identify mechanisms of exposure with response prevention (ERP) that predict short-term outcomes in CBT for obsessive compulsive disorder. We focused on exposure process variables derived from emotional processing theory (EPT) and inhibitory learning theory (ILT [22–25, 28]), and assessed different types of distress-related expectancy violation and habituation. Our results indicate that both habituation and distress-related expectancy violation during the first exposure have capacity to predict outcomes, depending on the outcome measure applied.

Regarding habituation parameters our analyses revealed that within-session habituation during the first standardized ERP (WSH_ERP1_) significantly predicted the percentage change on the Y-BOCS from t_1_ to t_20_. Thus, a stronger decline of subjective fear or distress during the first exposure session was associated with a stronger decrease of OCD symptoms after twenty sessions of CBT. This finding was consistent across two different operationalizations of within-session habituation. Regardless of whether the parameter was calculated as a difference score between the maximum SUD level and the ensuing minimum SUD level (WSH_ERP1_, Table 4, Fig. 3) or whether mixed-effect models were applied in order to extract random linear slopes of the SUD course across time (Slope_ERP1_, Supplementary Table 3), the first exposure within-session habituation remained a significant predictor.

However, neither within-session habituation during the second standardized ERP nor between-session habituation predicted percentage change on the Y-BOCS. Taken together, these findings are partially consistent with previous research that found WSH to be predictive for treatment outcome [39, 42]. However, Kircanski and Peris [43] did not find this association. One possible explanation refers to the operationalization of WSH: while Foa, Grayson [39] applied operationalizations comparable to the present study, Kircanski and Peris [43] assessed WSH as the decrease in distress across different exposure tasks within one session and not within the same task. Further, we failed to replicate an association between BSH and treatment outcome, which has been suggested by earlier studies [39, 41, 43]. Yet, we repeated the first ERP identically in order to assess BSH without possible contamination by new exposure tasks, which has not been done in previous studies. If BSH would indeed be a mechanism of action our strictly standardized study setup would be well suited to reveal its effect.

Despite its predictivity for percentage change on the Y-BOCS, WSH during the first exposure did not predict remission status (Table 5, Fig. 3). On the other hand, expectancy violation towards the end SUD score (EVend) in the first ERP session significantly predicted remission status at t_20_ (early remission, Table 5, Fig. 3), but not percentage change on the Y-BOCS (Table 4, Fig. 3). These results consistently indicate a positive relationship between remission status and lower experienced than expected SUDs at the end of the first ERP (Table 5, Fig. 3). The Odds Ratio of 2.03 (Table 5) indicates that the chance to remit early during treatment doubles if the discrepancy between expected and experienced SUDs rises by one unit. However, the same type of distress-related expectancy violation negatively predicted remission status if present during the identical repetition of the ERP task in the next session (Table 5, Fig. 3).

As EVend during the first exposure was the only significant predictor for early remission, an overestimation of fear expected for the end of exposure may represent a key measure of expectancy violation in OCD: achieving a surprise driven by a lower actual end distress level than was expected might initiate learning mechanisms connected with rapid achievement of subthreshold symptom severity. However, apparently this must take effect during the first ERP session for when repeating the session identically, the same mechanism tends to yield negative effects on remission status. This reversal of effects is surprising. But the negative association between early remission and distress-related expectancy violation in the second ERP session may be explained by an overestimation of fear in the second ERP session that might reflect insufficient learning from experience in the first ERP session.

Previous studies on OCD did not find significant relations between distress-related expectancy violation and treatment outcomes. However, these studies did not apply expectancy violation towards the end SUD score, but measured the difference between expected and actual maximum or average SUD scores [43, 46]. In the present study, similar parameters (EVmax, EVself) neither predicted outcome, but the difference between expected and actual fear levels at the very end of the exposure session did. Notably, remitters showed significantly stronger expectancy violation towards the end fear level in terms of overestimation. Hence, an overestimation of fear regarding the terminal point of exposure is associated with early remission.

Taken together, we found two significant predictors for treatment outcomes during the first exposure: while WSH predicted percentage change on the Y-BOCS, EVend predicted remission status. Although these predictors correlate moderately, our models consider both variables and demonstrate their differential capacity for prediction. In particular, a one-dimensional CFA model yielded inadequate fit indices, suggesting that the assessed parameters are not indicators for the same construct. In addition, our path model suggests that theoretically distinct variables relate differently to early remission on the one hand and percentage change on the other hand. As remission appears to reflect more sustainable change in OCD symptoms [6], it appears tempting to speculate that expectancy violation is of higher relevance for full recovery. However, it is also possible that initial within-session habituation induces processes of change that are slower and take somewhat longer to enable remission. Further insight is expected from future analyses of long-term outcome. Considering the present results, we assume that process variables derived from both EPT and ILT are related to outcomes of ERP in OCD.

Our data further suggest an extraordinary relevancy of the first exposure experience compared to an identical repetition. Therefore, planning and conducting the first ERP might be of particular importance, and should be optimized to allow both habituation and expectancy violation. Therapist might consider, for example, that the fear level expected for the situation is high enough to allow for noticeable violation. This may suggest to omit extensive cognitive interventions prior to exposure that might reduce the discrepancy between expected and actual outcome and to deepen reflection about the observed discrepancy after exposure [28]. Also, exposure tasks could be planned to last long enough for habituation to take place, for instance until fear levels have reduced significantly as it is often suggested in clinical practice. However, our study was not designed to investigate how to achieve or optimize expectancy violation or habituation, respectively.

In this study we focused on clinical indicators of habituation and distress-related expectancy violation assessed in manual-based CBT that can be assessed easily and conveniently during ERP. However, the discrepancy between expected and actual ability to tolerate distress could be an alternative measure that was not assessed in the present study. Moreover, the current analysis was restricted to short-term outcome after a first manualized phase of treatment and therefore the results are not readily transferrable to outcome at the end of treatment. Accordingly, the size of treatment effects was lower than the average effect size of outcome studies, which usually refer to complete treatments [3]. Follow-up analyses and future studies will have to show whether the current findings on outcome prediction by habituation and expectancy violation also hold for outcome assessments at post-treatment and follow-up time points. According to inhibitory learning theory, expectancy violation is expected to be especially beneficial for long-term outcomes (e.g., [25]).

Notably, the present study was done under naturalistic conditions and no experimental variation was applied. While effectiveness studies have advantages regarding generalization of finding to real-world conditions [70], the present study deviates from routine care treatment by applying a manual designed to assess clinical indicators during exposure. For example, therapists do not typically repeat the first exposure within few days in clinical practice. Further, the duration of ERP exercises is usually not fixed and exercises are often adaptively changed within the same ERP session. This was not the case in the present study because standardization appeared necessary to investigate mechanisms of exposure and response prevention. Thus, data was missing for exercises that were terminated prematurely. On the other hand, standardization was also limited in the present study as adherence was only controlled by checklist-based therapist ratings, but not by independent video-based ratings. Additionally, medication was not stable for all participants during the study period and the rather strict study protocol yielded a relatively large amount of missing data due to protocol violations. Moreover, the timing of the first ERP varied within the range of session five to 15 as a result of skipped optional or repeated mandatory manual contents. Despite a multitude of potential influences on outcome even during the first twenty therapy sessions in the present study, we were able to demonstrate potential impacts of theoretically founded clinical indicators by significant albeit small effects. Of course, correlational data do not permit firm conclusions on causality. However, the putative processes preceded the outcome assessment and empirical correlations correspond to theoretical assumptions. Nevertheless, further research is needed to investigate whether other variables like adherence, motivation or therapeutic alliance might explain the relationship between the process variables and outcome, and test a causal relationship using experimental methods, for example in randomized controlled trials. The present data can help to specify the target variables of experimental variations.

Although we found a significant correlation between expectancy violation and short-term outcome, the study might have been limited in detecting even larger effects. First, the therapeutic procedures were not optimized to maximally violate expectancies as suggested by proponents of inhibitory learning theory (e.g., applying multiple fear cues or a variable order of exposure tasks; see [71]). Second, we chose to focus on distress-related expectancy violation, although theoretical conceptions primarily suggest measuring the discrepancy between feared and actually occurring events [25, 28]. The selection was justified by clinical considerations and consistent with other OCD studies, but precludes conclusions on event related expectancies. Although the latter should be considered in a more comprehensive representation of inhibitory learning theory, the present data point to the utility of measuring expectancy violations concerning distress.

The relationship between distress-related expectancy violation and outcome also highlights a putatively prominent role of distress management in the maintenance of OCD symptoms. It has been suggested, for example, that reduced distress tolerance might contribute to the development or maintenance of OCD and other psychopathological symptoms (e.g., [72, 73]). Although distress tolerance and distress-related expectancy violation are distinct constructs, they may not be independent from each other. It appears possible, for example, that distress-related expectancy violation leads to changes in distress tolerance or reflects the individual’s pre-existing ability or willingness to tolerate distress to some degree. As we did not assess distress tolerance in the present study, future studies should investigate the relationship between distress-related expectancy violation and distress tolerance. In addition, the discrepancy between the expected and the experienced ability to *tolerate* distress should be captured as another facet of expectancy violation [47].

As for expectancy violation, conceptual issues might also be discussed for habituation. Although we found a relationship between within-session habituation and short-term reduction of OCD symptoms, it remains unclear whether habituation can be considered a mechanism of action during ERP or whether it should rather be considered an indicator of extinction [28]. Recent research made efforts to clarify the exact conditions of extinction learning in OCD and its relation to therapy outcome [14, 74, 75].

Importantly, our data derive from routine clinical procedures and the indicators of habituation and distress-related expectancy violation can be assessed and computed easily by therapists. This is highlighted by virtually no differences in regression models considering minimum versus maximum scores on one hand, and random effects linear slopes on the other hand (Supplementary Tables 3 and 4). Thus, our findings might be of high clinical utility and external validity. This is also true because ERP in routine clinical practice is usually conducted without the assessment of psychophysiological measures like skin conductance response, heart rate, or others. However, a comprehensive scientific evaluation of constructs needs to integrate this level of measurement, especially in the case of emotional processing theory. Therefore, future studies should also examine the predictive value of psychophysiological measures for treatment outcome, and its relation to subjective fear reports.

## Conclusions

Exposure and response prevention has proven to be the central treatment element of CBT for OCD. The course of the mean Y-BOCS-self-rating score in the present study underlines this notion since mean symptom severity remains unchanged until the time immediately before the first ERP and thereafter declines significantly (Fig. 2). Theoretical approaches presume habituation or expectancy violation as important mechanisms of change in exposure-based therapy. The processes underlying ERP, however, are rarely specified empirically. In the present study, we provide first data from exposure-based CBT with a large sample of 110 adult patients with OCD. Notably, we are the first to find evidence of a relationship between distress-related expectancy violation and outcome in OCD. However, our results are reconcilable with both theoretical approaches. If our findings can be confirmed by future research including experimental approaches, they may guide training, implementation and evaluation of exposure-based treatment of OCD.

## Supplementary Information

**Additional file 1: Supplementary Table 1**. Comorbid mental disorders and medication status at admission (t_0_).

**Additional file 2: Supplementary Table 2**. Intercorrelations among exposure process variables.

**Additional file 3: Supplementary Table 3**. Multiple regression model predicting percentage change of the Y-BOCS score from t_1_ to t_20_ using random effects linear slopes estimating WSH.

**Additional file 4: Supplementary Table 4**. Logistic regression model predicting remission status at t_20_ using random effects linear slopes estimating WSH.

**Additional file 5: Supplementary Table 5**. Standardized factor loadings for one-dimensional confirmatory factor analysis model.

## Data Availability

The datasets used and analyzed during the current study are available from the corresponding author on reasonable request.

## Abbreviations

OCD: Obsessive-compulsive disorders
Y-BOCS: Yale-Brown Obsessive-Compulsive Scale
ERP: Exposure and response prevention
CBT: Cognitive behavioral therapy
EPT: Emotional processing theory
ILT: Inhibitory learning theory
SUD: Subjective Units of Distress
WSH: Within-session habituation
BSH: Between-session habituation
CY-BOCS: Children’s Yale-Brown Obsessive-Compulsive Scale
MADRS: Montgomery-Åsberg Depression Rating Scale
GAF: Global Assessment of Functioning
OCI-R: Obsessive Compulsive Inventory – Revised
BDI-II: Beck Depression Inventory II
BSI: Brief Symptom Inventory
t_1_: First therapy session
t_20_: Twentieth therapy session
ERP1: First session with exposure and response prevention
ERP2: Second session with exposure and response prevention
EVmax: Expectancy violation towards the maximum SUD score
EVend: Expectancy violation towards the end SUD score
EVself: Direct self-rating of expectancy violation towards the maximum SUD score
SEC: ERP-related self-efficacy change
CFA: Confirmatory factor analysis
