# Supplementary material for: Mechanisms of exposure and response prevention in obsessive-compulsive disorder: effects of habituation and expectancy violation on short-term outcome in cognitive behavioral therapy
Source: BMC Psychiatry. 2022 Jan 27;22:66. doi: 10.1186/s12888-022-03701-z (PMC8793233; doi:10.1186/s12888-022-03701-z)
Supplement: Supplementary file 1 — Additional file 1: Supplementary Table 1. Comorbid mental disorders and medication status at admission (t0). [file 12888_2022_3701_MOESM1_ESM.docx]

**Supplementary Table 1.**

Comorbid Mental Disorders and Medication Status at Admission (t_0_)

|  | *n* | % |
| --- | --- | --- |
| Number of comorbid mental disorder |  |  |
| 0 | 22 | 20.0 |
| 1 | 57 | 51.8 |
| 2 | 19 | 17.3 |
| 3 | 9 | 8.2 |
| 4 | 3 | 2.7 |
| Diagnosis of comorbid mental disorder |  |  |
| remitted disorder due to psychoactive substance use | 3 | 2.7 |
| current affective disorder | 43 | 39.1 |
| remitted affective disorder | 31 | 28.2 |
| any anxiety disorder | 38 | 34.5 |
| eating disorder | 2 | 1.8 |
| nonorganic sleep disorder | 1 | 0.9 |
| personality disorder | 7 | 6.4 |
| tic disorder | 3 | 2.7 |
| other specified disorder with onset in childhood and adolescence | 1 | 0.9 |
| Psychotropic medications |  |  |
| none | 62 | 56.4 |
| antidepressants |  |  |
| citalopram | 1 | 0.9 |
| escitalopram | 12 | 10.9 |
| fluoxetine | 8 | 7.3 |
| fluvoxamine | 3 | 2.7 |
| paroxetine | 8 | 7.3 |
| sertraline | 5 | 4.5 |
| milnacipran | 1 | 0.9 |
| venlafaxine | 4 | 3.6 |
| mirtazapine | 3 | 2.7 |
| clomipramine | 2 | 1.8 |
| trimipramine | 3 | 2.7 |
| moclobemide | 2 | 1.8 |
| antipsychotics |  |  |
| amisulpride | 1 | 0.9 |
| olanzapine | 1 | 0.9 |
| quetiapine | 3 | 2.7 |
| risperidone | 3 | 2.7 |
| flupentixol | 1 | 0.9 |
| pipamperone | 1 | 0.9 |
| others |  |  |
| lorazepam (if required) | 2 | 1.8 |
| methylphenidate | 1 | 0.9 |
