# Supplementary material for: Mechanisms of exposure and response prevention in obsessive-compulsive disorder: effects of habituation and expectancy violation on short-term outcome in cognitive behavioral therapy
Source: BMC Psychiatry. 2022 Jan 27;22:66. doi: 10.1186/s12888-022-03701-z (PMC8793233; doi:10.1186/s12888-022-03701-z)
Supplement: Supplementary file 2 — Additional file 2: Supplementary Table 2. Intercorrelations among exposure process variables. [file 12888_2022_3701_MOESM2_ESM.docx]

**Supplementary Table 2.**

*Intercorrelations among exposure process variables.*

|  | WSH_ERP1_ | WSH_ERP2_ | BSH | EVmax_ERP1_ | EVmax_ERP2_ | EVend_ERP1_ | EVend_ERP2_ | EVself_ERP1_ | EVself_ERP2_ | SEC_ERP1_ | SEC_ERP2_ |
| --- | --- | --- | --- | --- | --- | --- | --- | --- | --- | --- | --- |
| WSH_ERP1_ | - |  |  |  |  |  |  |  |  |  |  |
| WSH_ERP2_ | .57*** | - |  |  |  |  |  |  |  |  |  |
| BSH | .27** | .02 | - |  |  |  |  |  |  |  |  |
| EVmax_ERP1_ | -.00 | .09 | -.13 | - |  |  |  |  |  |  |  |
| EVmax_ERP2_ | -.04 | -.11 | .51*** | .13 | - |  |  |  |  |  |  |
| EVend_ERP1_ | .41*** | .27** | .09 | .42*** | -.00 | - |  |  |  |  |  |
| EVend_ERP2_ | .03 | .36*** | .32*** | .06 | .50*** | .23* | - |  |  |  |  |
| EVself_ERP1_ | .12 | .14 | .04 | .55*** | .07 | .34*** | .14 | - |  |  |  |
| EVself_ERP2_ | .07 | .29** | .34*** | .09 | .41*** | .04 | .43*** | .16 | - |  |  |
| SEC_ERP1_ | .06 | -.01 | -.10 | .02 | .07 | -.07 | -.10 | -.05 | -.02 | - |  |
| SEC_ERP2_ | .01 | .00 | .05 | -.21* | -.01 | -.07 | .08 | -.03 | -.07 | .08 | - |

*Note*. ERP1 = first standardized exposure with response prevention; ERP2 = second standardized exposure with response prevention; WSH = within-session habituation; BSH = between-session habituation; EVmax = expectancy violation towards the maximum SUD score; EVend = expectancy violation towards the end SUD score; EVself = direct self-rating of expectancy violation towards the maximum SUD score; SEC = self efficacy change; * *p* < .05; ** *p* < .01; *** *p* < .001
