# Supplementary material for: Mechanisms of exposure and response prevention in obsessive-compulsive disorder: effects of habituation and expectancy violation on short-term outcome in cognitive behavioral therapy
Source: BMC Psychiatry. 2022 Jan 27;22:66. doi: 10.1186/s12888-022-03701-z (PMC8793233; doi:10.1186/s12888-022-03701-z)
Supplement: Supplementary file 3 — Additional file 3: Supplementary Table 3. Multiple regression model predicting percentage change of the Y-BOCS score from t1 to t20 using random effects linear slopes estimating WSH. [file 12888_2022_3701_MOESM3_ESM.docx]

**Supplementary Table 3.**

*Multiple regression model predicting percentage change of the Y-BOCS score from t_1_ to t_20_ using random effects linear slopes estimating WSH.*

| Coefficient | B (SE) | β | 95% CI for β | p |
| --- | --- | --- | --- | --- |
| Constant | -0.15 (14.93) |  |  | .992 |
| Y-BOCS score t_1_ | 0.75 (0.43) | 0.16 | -0.11 - 1.60 | .087 |
| Slope_ERP1_ | -0.25* (0.10) | -0.32 | -0.45 - -0.04 | .020 |
| EVmax_ERP1_ | 2.65 (1.94) | 0.17 | -1.20 - 6.51 | .175 |
| EVend_ERP1_ | 0.95 (1.15) | 0.10 | -1.32 - 3.23 | .407 |
| EVself_ERP1_ | 0.10 (1.34) | 0.01 | -2.56 - 2.77 | .938 |
| SEC_ERP1_ | 0.30 (1.14) | 0.02 | -1.96 - 2.56 | .793 |
| Slope_ERP2_ | 0.22 (0.13) | 0.25 | -0.03 - 0.47 | .088 |
| EVmax_ERP2_ | 0.18 (1.99) | 0.01 | -3.77 - 4.13 | .929 |
| EVend_ERP2_ | 0.38 (1.53) | 0.04 | -2.65 - 3.41 | .806 |
| EVself_ERP2_ | -1.07 (1.40) | -0.08 | -3.86 - 1.71 | .446 |
| SEC_ERP2_ | 2.80 (1.57) | 0.17 | -0.32 - 5.92 | .078 |
| BSH | 0.72 (2.10) | 0.04 | -3.45 - 4.89 | .732 |

*Note*. Y-BOCS = Yale-Brown Obsessive-Compulsive Scale interview; ERP1 = first standardized exposure with response prevention; ERP2 = second standardized exposure with response prevention; Slope = within-session habituation as estimated by random effects linear slopes; BSH = between-session habituation; EVmax = expectancy violation towards the maximum SUD score; EVend = expectancy violation towards the end SUD score; EVself = direct self-rating of expectancy violation towards the maximum SUD score; SEC = self efficacy change; * *p* < .05; ** *p* < .01; *** *p* < .001
