# Supplementary material for: Mechanisms of exposure and response prevention in obsessive-compulsive disorder: effects of habituation and expectancy violation on short-term outcome in cognitive behavioral therapy
Source: BMC Psychiatry. 2022 Jan 27;22:66. doi: 10.1186/s12888-022-03701-z (PMC8793233; doi:10.1186/s12888-022-03701-z)
Supplement: Supplementary file 4 — Additional file 4: Supplementary Table 4. Logistic regression model predicting remission status at t20 using random effects linear slopes estimating WSH. [file 12888_2022_3701_MOESM4_ESM.docx]

**Supplementary Table 4.**

*Logistic regression model predicting remission status at t_20_ using random effects linear slopes estimating WSH.*

| Coefficient | B (SE) | exp b (OR) | 95% CI for OR | p |
| --- | --- | --- | --- | --- |
| Constant | 1.44 (2.60) |  |  | .578 |
| Y-BOCS score t_1_ | -0.26*** (0.07) | 0.77 | 0.65 - 0.88 | < .001 |
| Slope_ERP1_ | 0.02 (0.02) | 1.02 | 0.99 - 1.06 | .159 |
| EVmax_ERP1_ | 0.08 (0.41) | 1.09 | 0.48 - 2.48 | .836 |
| EVend_ERP1_ | 0.76** (0.24) | 2.14 | 1.42 - 3.73 | .002 |
| EVself_ERP1_ | -0.07 (0.25) | 0.93 | 0.56 - 1.55 | .771 |
| SEC_ERP1_ | -0.08 (0.26) | 0.92 | 0.52 - 1.46 | .745 |
| Slope_ERP2_ | -0.01 (0.02) | 0.99 | 0.95 - 1.03 | .690 |
| EVmax_ERP2_ | 0.49 (0.37) | 1.63 | 0.84 - 3.69 | .187 |
| EVend_ERP2_ | -0.51 (0.26) | 0.60 | 0.34 - 0.98 | .052 |
| EVself_ERP2_ | -0.30 (0.26) | 0.74 | 0.43 - 1.22 | .255 |
| SEC_ERP2_ | 0.25 (0.27) | 1.29 | 0.77 - 2.28 | .341 |
| BSH | 0.38 (0.37) | 1.47 | 0.69 - 3.08 | .302 |

*Note*. OR = Odds Ratio; Y-BOCS = Yale-Brown Obsessive-Compulsive Scale interview; ERP1 = first standardized exposure with response prevention; ERP2 = second standardized exposure with response prevention; Slope = within-session habituation as estimated by random effects linear slopes; BSH = between-session habituation; EVmax = expectancy violation towards the maximum SUD score; EVend = expectancy violation towards the end SUD score; EVself = direct self-rating of expectancy violation towards the maximum SUD score; SEC = self efficacy change; * *p* < .05; ** *p* < .01; *** *p* < .001
