# Supplementary material for: Mechanisms of exposure and response prevention in obsessive-compulsive disorder: effects of habituation and expectancy violation on short-term outcome in cognitive behavioral therapy
Source: BMC Psychiatry. 2022 Jan 27;22:66. doi: 10.1186/s12888-022-03701-z (PMC8793233; doi:10.1186/s12888-022-03701-z)
Supplement: Supplementary file 5 — Additional file 5: Supplementary Table 5. Standardized factor loadings for one-dimensional confirmatory factor analysis model. [file 12888_2022_3701_MOESM5_ESM.docx]

**Supplementary Table 5.**

*Standardized factor loadings for one-dimensional confirmatory factor analysis model.*

| Indicator | factor loading |
| --- | --- |
| WSH_ERP1_ | 0.164 |
| BSH | -0.027 |
| EVmax_ERP1_ | 0.775*** |
| EVend_ERP1_ | 0.545*** |
| EVself_ERP1_ | 0.692*** |
| SEC_ERP1_ | 0.017 |

*Note*. ERP1 = first standardized exposure with response prevention; WSH = within-session habituation; BSH = between-session habituation; EVmax = expectancy violation towards the maximum SUD score; EVend = expectancy violation towards the end SUD score; EVself = direct self-rating of expectancy violation towards the maximum SUD score; SEC = self efficacy change; * *p* < .05; ** *p* < .01; *** *p* < .001
